# Is the closest health facility the one used in pregnancy care-seeking? A cross-sectional comparative analysis of self-reported and modelled geographical access to maternal care in Mozambique, India and Pakistan

**DOI:** 10.1186/s12942-020-0197-5

**Published:** 2020-02-03

**Authors:** Liberty Makacha, Prestige Tatenda Makanga, Yolisa Prudence Dube, Jeffrey Bone, Khátia Munguambe, Geetanjali Katageri, Sumedha Sharma, Marianne Vidler, Esperança Sevene, Umesh Ramadurg, Umesh Charantimath, Amit Revankar, Peter von Dadelszen

**Affiliations:** 1 Faculty of Science and Technology, Surveying and Geomatics, Midlands State University, Gweru, Zimbabwe; 2 Department of Obstetrics and Gynaecology, University of British Columbia, Vancouver, Canada; 3 Centro de Investigação EM Saúde de Manhiça, Manhiça, Mozambique; 4 Faculty of Medicine, Eduardo Mondlane University, Maputo, Mozambique; 5 Department of Obstetrics and Gynaecology, S. Nijalingappa. Medical College and H.S.K. Hospital & Research Centre, Bagalkot, Karnataka India; 6 Department of Community Medicine, S. Nijalingappa. Medical College and H.S.K. Hospital & Research Centre, Bagalkot, Karnataka India; 7 Department of Community Medicine, K.L.E. University’s Jawaharlal Nehru Medical College, Belgaum, Karnataka India; 8 Department of Women and Children’s Health, School of Life Course Sciences, King’s College London, London, UK

**Keywords:** Potential access, Realised access, Bland–Altman Index, Fixed bias, Limits of agreement, Proportional bias

## Abstract

**Background:**

Travel time to care is known to influence uptake of health services. Generally, pregnant women who take longer to transit to health facilities are the least likely to deliver in facilities. It is not clear if modelled access predicts fairly the vulnerability in women seeking maternal care across different spatial settings.

**Objectives:**

This cross-sectional analysis aimed to (i) compare travel times to care as modelled in a GIS environment with self-reported travel times by women seeking maternal care in Community Level Interventions for Pre-eclampsia: Mozambique, India and Pakistan; and (ii) investigate the assumption that women would seek care at the closest health facility.

**Methods:**

Women were interviewed to obtain estimated travel times to health facilities (R). Travel time to the closest facility was also modelled (P) (closest facility tool (ArcGIS)) and time to facility where care was sought estimated (A) (route network layer finder (ArcGIS)). Bland–Altman analysis compared spatial variation in differences between modelled and self-reported travel times. Variations between travel times to the nearest facility (P) with modelled travel times to the actual facilities accessed (A) were analysed. Log-transformed data comparison graphs for medians, with box plots superimposed distributions were used.

**Results:**

Modelled geographical access (P) is generally lower than self-reported access (R), but there is a geography to this relationship. In India and Pakistan, potential access (P) compared fairly with self-reported travel times (R) [P (H_0_: Mean difference = 0)] < .001, limits of agreement: [− 273.81; 56.40] and [− 264.10; 94.25] respectively. In Mozambique, mean differences between the two measures of access were significantly different from 0 [P (H_0_: Mean difference = 0) = 0.31, limits of agreement: [− 187.26; 199.96]].

**Conclusion:**

Modelling access successfully predict potential vulnerability in populations. Differences between modelled (P) and self-reported travel times (R) are partially a result of women not seeking care at their closest facilities. Modelling access should not be viewed through a geographically static lens. Modelling assumptions are likely modified by spatio-temporal and/or socio-cultural settings. Geographical stratification of access reveals disproportionate variations in differences emphasizing the varied nature of assumptions across spatial settings.

*Trial registration* ClinicalTrials.gov, NCT01911494. Registered 30 July 2013, https://clinicaltrials.gov/ct2/show/NCT01911494

## Background

Globally geographical access to maternal care is one key driver of health reform efforts and remains an important indicator of quality of maternal health care [1]. Travel times to health services especially primary care is used as a parameter for health care delivery assessment and health policy evaluation in most parts of the world [2]. Lack of empirical data on travel time to care warrants the use of Geographical Information Systems (GIS) in modelling access as either travel time or travel distance [3, 4]. This approach bridges data gaps arising from inefficient or costly data collection procedures. Assumptions which go into modelling access impact heavily on the results and recommendations from access modelling. It is therefore imperative to validate the extent of validity of assumptions in access modelling across different spatial settings. In a study in Mozambique [5] assumed that women would seek care at their closest facilities.

There is also substantial adjunct literature on the operationalisation of the term geographical access to care, rendering inconsistencies in the definition and understanding of the term “access to care”. In this study, our operational definition of access to care, is travel times to care facilities during pre- and/or postnatal care through the referral system triggered through the CLIP trials, from an origin point; a definition which conforms more to addressing the second delay in access to care. References [6, 7] articulates the three delays as: delays at home prior to deciding to seek care (the first delay); delays in finding and managing transport to a facility (the second delay); and delays in receiving appropriate treatment once at the facility (the third delay).

Identifying differences between modelled geographical access and self-reported travel times is essential (1) methodologically to inform selection of techniques to measure access and (2) substantively to understand where disparities in access exist and how to intervene accordingly, especially where predicting population vulnerabilities is key.

In studies which have attempted to validate outcomes from different measures of access [8–10] different methodological approaches have been used. Among the methods that have been used for method outcomes comparison are graphical correlation and regression [10], Spearman’s Rank correlation coefficient [9] or grouped proportions in stratified subgroupings [8]. For continuous scale measurements, validation of outcome measurements by two different techniques are either by the Bland–Altman method of differences or least products regression analysis. Researchers who have chosen not to use the former method argue that it does not distinguish adequately between fixed and proportional bias. However, the technique has been proven to be more robust, especially in instances where prior limits of agreement are available. Several studies agree that the Pearson product–moment correlation coefficient (r) is valueless as a test for bias [11, 12], even though the technique has been used in some studies, for example in [9].

The objectives of this study are to compare realised access (R) as self-reported by women accessing maternal services in CLIP trials Mozambique, India and Pakistan with modelled geographical access to care (P).^1^ We challenge the assumption that access to maternal care is to the closest health facility.

## Methods

### Study design and setting

**Fig. 1:**
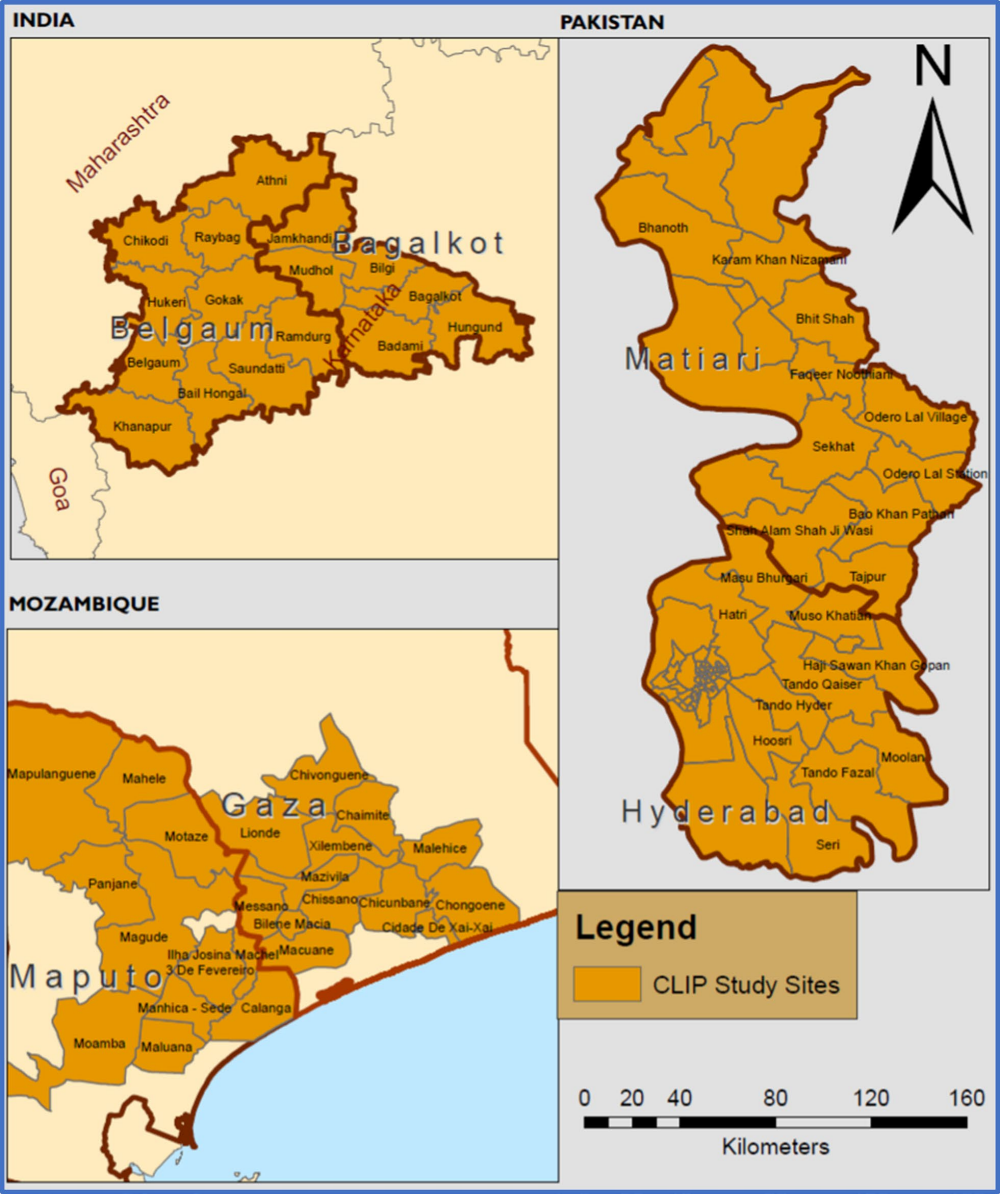
CLIP Mozambique, India and Pakistan study sites

This study was conducted as part of the Community Level Interventions for Pre-eclampsia (CLIP) trials. The study is a population-level cross sectional secondary analysis from the CLIP cluster randomized controlled trials [13] that introduces evidence-based interventions applied primarily at the community level to reduce maternal and perinatal mortality and morbidity in the intervention clusters resulting from the failure to identify and manage pre-eclampsia.. The study draws evidence from Maputo and Gaza provinces southern Mozambique, Karnataka, India and Sindh, Pakistan (Fig. 1). The trials were designed to address the excess maternal and perinatal mortality in low- and middle-income countries (LMICs) with participants all from a non-masked parallel assignment intervention model.

### Study population

**Fig. 2:**
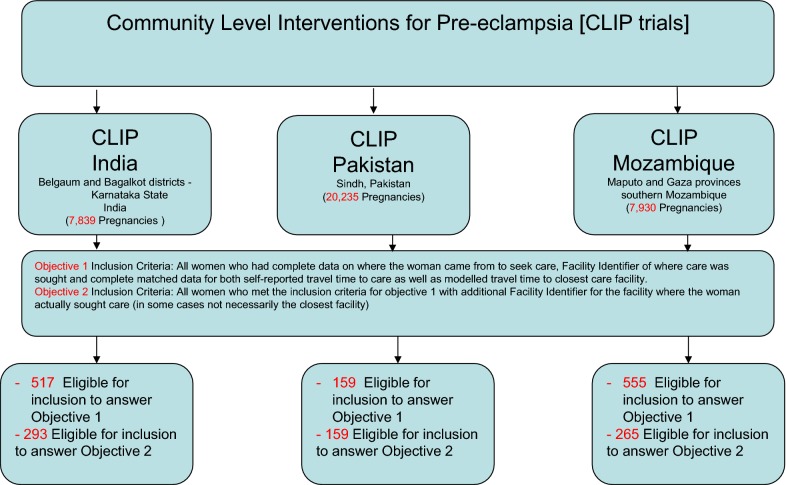
Study sites with final participating samples per study site

Our study population includes all women aged 15–49 years who participated in the CLIP trials between September 2013 and May 2018. Samples of pregnant or postpartum women with complete data on where the woman came from to access care, where care was sought, as well as paired data on both self-reported and geographically modelled travel time to care facilities in the three study sites were included in the study. The final participating samples included into the study were 555 women in Mozambique, 517 in India and 159 pregnant women in Pakistan. These women qualified for inclusion to answer the first objective of the study. Of these, 265 women in Mozambique, 293 women in India and all the 159 women in Pakistan qualified to answer the second objective of the study. Figure 2 below shows the inclusion/exclusion sample flow in this analysis.

## Procedure

### Overview

This analysis comprises evaluating estimated travel times to care facilities as self-reported by women from both the intervention and control clusters of the CLIP trials. For each woman self-reporting travel time to care, we modelled her potential access to care as a function of travel time, accounting for the effects of precipitation on access [5]. In addition, we evaluated closest facilities to the women’s place of residence and compared the travel times to these facilities with travel times to the facilities where the women sought care.

### Summary descriptions of the different methods of estimating travel time to care

Our parameters for this analysis were realised access (R), modelled travel time (P) and time to the facility where the woman sought care (A). Realised access is defined as self-reported travel time by pregnant women from their homestead (village) to the facility through the referral chain. Modelled travel time is defined as the total travel time from each woman’s village centre, to the closest health facility along the least cost surface. Travel time to facility where care was sought on the other hand, is the total time taken along the least cost surface from the woman’s village/or in cases where she would have migrated to a relative’s place, from that reported place to the facility where she was attended.

### Realised access (self-reported travel times to care)—method R

Using facility surveillance and regular surveillance data centrally administered from the University of British Columbia (UBC) Canada,^2^ for the three CLIP study sites, we evaluated migration patterns in transiting to care, mechanisms of accessing care as well as the estimated travel time to the care facilities as self-reported by the woman. In Mozambique, walking, *txvova* (hand pulled cart), and bicycling were considered walking modes to accessing care while motor bike and vehicle were considered driving options to care. In India and Pakistan ambulance, ‘hired vehicle’ and ‘own vehicle’ were categorised as driving mode of accessing care. Accessing care using the village vehicle was considered a public transport mode of accessing care.

Self-reported travel times to the facilities where women accessed maternal care were extracted from the CLIP surveillance data for each site. An indicator of travel time was deduced from questions asking either the estimated travel time to the point of care or in some instances through a direct deduction given the start time and the end time of transiting to care. Only women who had a reported travel time to the facility where care was sought were included in the study.

### Potential access—modelled travel time (method P)

Potential access denotes travel times to health facilities modelled in a GIS environment [5], with the key assumption that facility utilisation was at the closest facility to the woman’s place of residence and that travel time is impeded by seasonal variations. Precipitation data was downloaded from fewsnet data portal (https://earlywarning.usgs.gov/fews/). We classified precipitation rasters into dry and wet categories. We considered all precipitation under 2 mm of rain as dry and all precipitation greater or equal to 2 mm as wet. Vectorised precipitation data was appended to the network data by date of accessing care for each woman in modelling access, a process which accounts for the seasonal effect on access. Travel times to the closest facility was computed for each woman, accounting for road type, probable travel speeds along the road segments as well as precipitation status on the date of accessing care.

### Travel time to facility where care was sought (method A)

Method A represent travel times along the least cost route between the known start node, village centre of woman or some known and geographically defined health facility to the health facility where the woman actually went for care seeking using network data either digitized and/or downloaded from OpenStreetMap (https://www.openstreetmap.org). ArcGIS route layer calculator^3^ was used to calculate total travel time from village centroid to facility where care was sought for each woman who met the inclusion criteria. We compared this travel time with the time to the closest facility to validate the assumption that care seeking is to the closest facility.

### Statistical analysis

We present an analysis comparing GIS modelled geographical access to care and self-reported travel times to maternal health services using the Bland–Altman Statistical Analysis in MedCalc.

The study analyses the extent of variation of differences between modelled travel times and self-reported travel times using Bland–Altman Plots where the Bland–Altman Index $({\text{BAI}}) = {\text{Average}} \left( \frac{{\text{(P}} - {\text{R)}}}{{\text{(P + R)/2}}} \right)$, with 95% confidence intervals for the mean difference (a bias indicator) as well as the limits of agreement to determine the ranges in which the true value may lie, because different samples from the same populations might yield slightly different descriptives. Our choice of benchmarking analysis on potential access was informed by the fact that self-reported travel times are notoriously known to be inaccurate from previous studies [14].

We also present an analysis comparing the variation in the differences between travel times to the closest facilities and travel times to the facilities where care was sought. The analysis of the variations between modelled travel times to the closest facilities of each woman’s village of origin with modelled travel times to the facilities where the women went for care seeking was done as a measure of validating the assumption that women would seek care at their closest facilities. The study used log-transformed data comparison graphs for medians, with box plot superimposed distributions of all data points. Our sub-sample for validating the assumption of care seeking at the closest facility was further informed by the availability of geocoded facilities data for facilities used by the women.

## Results

### Comparison of modelled geographical access with self-reported travel times to care

The study provides evidence that modelled geographical access (P) is generally lower than self-reported access (R), but this relationship is modified by geography. The table below (Table 1) compares modelled geographical access with self-reported travel times to care facilities in the three CLIP study sites.

**Table 1:** Comparison of potential access with self-reported travel time to care—how different are the two methods

	Mean of both methods [95% CI]	*P* (H_0_: Mean = 0)	Standard deviation	Lower limit [95% CI of lower limit]	Upper limit [95% CI of lower limit]	Regression equation
Mozambique	6.35 [− 5.98;18.68]	0.3113	98.78	(187.26) [− 208.36; − 166.16]	199.96 [178.86;221.06]	y = − 5.37 + 10.22 x
India	(108.71) [− 115.98; − 101.43]	<.0001	84.24	(273.81) [− 286.25; − 261.36]	56.40 [43.95; 68.84]	y = − 104.31 + − 2.81x
Pakistan	(84.92) [− 99.24; − 70.60]	<.0001	91.42	(264.10) [− 288.62; − 239.57]	94.25 [69.73; 118.77]	y = − 92.99 + 16.63x

**Fig. 3:**
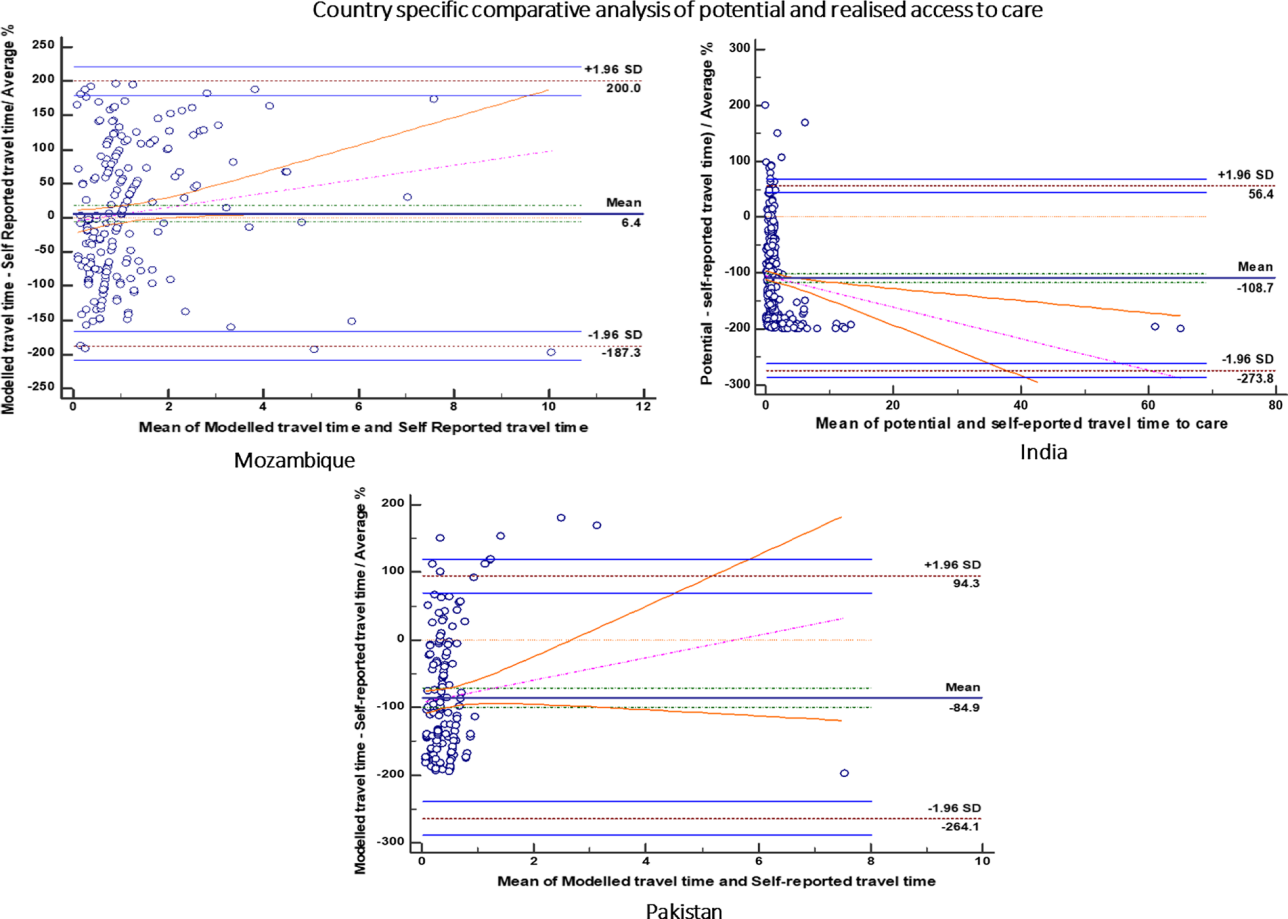
Country specific comparative analysis of potential and realised access to care

The differences between modelled geographical access (P) and self-reported access to care (R) are expressed as percentages of the mean of modelled geographical access and self-reported access to care (proportionally to the magnitude of the measurements). Table 1 (above) read with Fig. 3 (below) summarises the comparison of modelled geographical access with self-reported travel time to carew (Fig. 3) shows the Bland–Altiman plots comparing the two methods in the three study sites.

### Differences between modelled geographical access and self-reported access to care

In Mozambique, the differences between modelled travel times to care (P) and self-reported travel times to care (R) were statistically not significant [P (H_0_: Mean = 0)] = 0.31. Women self-reported travel times which were significantly lower than modelled travel times [Mean percentage difference = 6.35]. Mean differences were within the limits of agreement, implying that the two methods generally agree and may be used interchangeably for the sample under analysis. The limits of acceptability at 95% certainty were [− 208.36; 221.06].

The regression equation for the mean differences between modelled travel times and self-reported travel times to health care had a slope coefficient of 10.22 (P = 0.049 and 95% Confidence Interval of [0.06; 20.38]) and residual effect of − 5.37 with increasing differences in methods as mean travel time increases.

In India, self-reported travel times were significantly higher than modelled travel times [P value < 0.001]. The results show a generally increasing nett negative difference (P–R) with increasing mean travel time. The limits of agreement for the percentage differences were [− 286.25; 118.77], 95% Confidence Interval for the lower limit = [− 286.25; − 261.36] and 95% Confidence Interval for the Upper limit = [43.95;68.84]. The regression equation for the differences between modelled travel times and self-reported travel times to health care for the sample under study had a statistically significant slope coefficient of − 2.81 (P = 0.002 and 95% Confidence Interval of [− 4.55; − 1.07]).

In contrast to India and Mozambique, the differences between modelled travel times to care and self-reported travel times to care in Pakistan were statistically significant [P (H_0_: Mean = 0)] < 0.001. Women self-reported travel times which were significantly higher than modelled travel time [Mean percentage difference = − 84.92]. Most of the observations fell within the limits of agreement, implying that the two methods agree and may be used interchangeably (Limits of acceptability at 95% certainty = [− 288.62; 118.77]), with some systematic positive difference outliers.

The regression equation for the differences between modelled travel times and self-reported travel times to health care for the sample under study had a slope coefficient of 16.63 (P = 0.13 and 95% Confidence Interval of [− 4.82; 38.08] indicating a widening nett difference between the two methods.

The widening limits of agreement for the mean differences indicate decreasing reliability on the measure of differences, with increasing mean travel times. Therefore, model acceptability in Pakistan is highest for lower mean travel times.

### Do women seek care at the closest facilities?

In all three countries women do not necessarily seek care at the closest facilities [50.2%, 94.6% and 94.3%] in Mozambique, India and Pakistan respectively. This is also emphasized by the fact that the distributions of travel times to care facilities (A) were generally higher than modelled travel time to the closest facility. The distribution of travel times from the village centre to the closest facility (modelled travel time) and time from the village centre to the facility where care was sought (travel time to the facility where the woman sought care) are shown in the table below (Table 2).

**Table 2:** Comparison of distributions of modelled travel time with travel time to facility where care was sought

CLIP site	Travel time indicator	Modelled travel time	Time to facility where care was sought
Mozambique	Lowest value	0.04	0.04
	Highest value	8.08	26.81
	Median	0.52	1.00
	25th percentile	0.29	0.46
	75th percentile	1.13	3.50
India	Lowest value	0.01	0.13
	Highest value	3.83	26.01
	Median	0.19	1.09
	25th percentile	0.05	0.66
	75th percentile	0.38	1.92
Pakistan	Lowest value	0.01	0.12
	Highest value	5.77	19.15
	Median	0.15	0.44
	25th percentile	0.07	0.32
	75th percentile	0.32	0.74

**Fig. 4:**
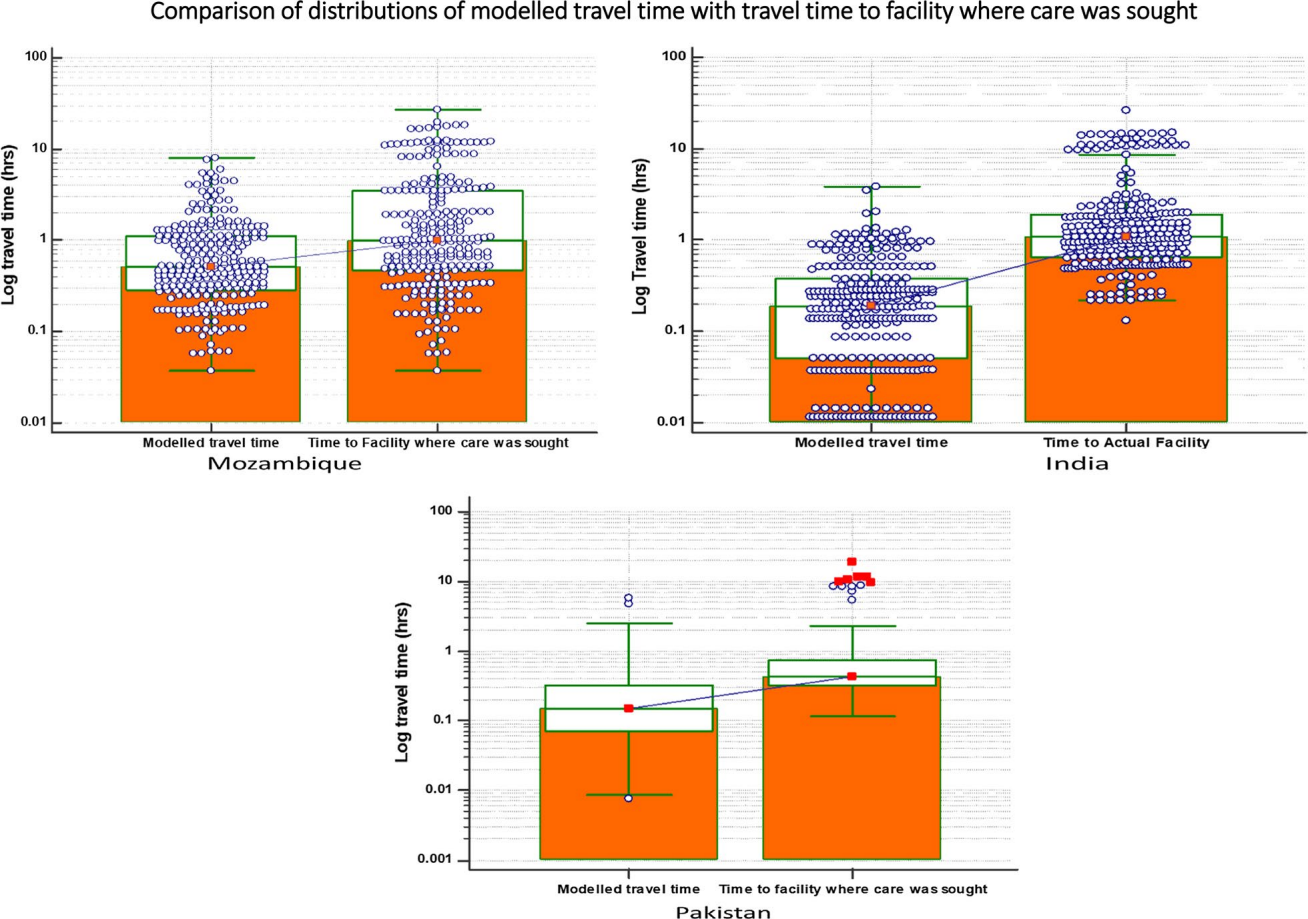
Comparisons of distributions of modelled travel time with travel time to facility where care was sought

Women generally did not seek care at the closest facility [Mozambique (median log modelled travel time [0.58] < median log of travel time to actual facility [0.999], India (median log of modelled travel time (0.19) < median Log of travel time to actual facility (1.09), Pakistan (median Log of modelled travel time (0.15) < median log of travel time to actual facility (0.44)). The distributions for actual travel times to care facilities (Fig. 4) were generally more positive than those for modelled travel times to care facilities. The midspread of modelled travel times to travel times to facilities where care was sought were Mozambique [0.84:3.03], India [0.33:1.27] and Pakistan [0.25:1.27].

## Discussion

### Summary of findings

Our analysis comparing modelled geographical access to care with self-reported travel times to care facilities, revealed generally acceptable levels of agreement between modelled travel time to health care facilities and self-reported travel time to care facilities with modelled geographical access generally lower than self-reported access. In Mozambique, however, the mean differences between the two measures of access were significantly different from 0. In accounting for access in a way that correlates well with reported access to care, modelled access may be as good as self-reported access Differences in the levels of agreement between the two methods of quantifying access may be partly explained by the spatio-temporal and/or socio-cultural differences between study settings. The study provides evidence that assumptions which went into modelling access to care should have accounted for other spatio-temporal differences that impede access to varying extents across different settings. This includes the changing nature of the health state of the woman seeking care [15] as well as other influences of the physical environment, such as distance to urban centres, limited transportation options, travel and delivery costs [16]. The current models under consideration did not account for effects of topography on access.

The study pointed out that to a larger extent, women would not seek care at facilities closest to their place of residence. A sizable proportion of women would seek care at other facilities not necessarily close to their residences. This is possibly motivated by factors such as kind and cost of services offered.

There is also a strong need to challenge the definition of “closest facilities” especially in different contexts, given that maternity in general and the perinatal period in particular is such a dynamic period, as women migrate between their homes, their husbands homes and their mother’s or in-laws homes as a function of their fertility pattern. Therefore, a definition of “closest’’ in all evaluation procedures should not be viewed statically, but rather with this dynamism in mind.

Since the majority of women did not access facilities closest to their place of residence this suggests that travel time to the closest facility may not serve as an adequate proxy for health facility utilization studies especially for geographically abundant services where the options are more and service consumption is predicted by other factors, including demand at the facility. The travel time metric which does not take demand at the facility into consideration, potentially may not be a full proxy for observed relationships and hence results should be interpreted within these confines.

### Strengths

Our measure of access, as a function of travel times to care, will do well in defining accessibility especially in Low to middle income economies where access should not be viewed as directly proportional to travel distances. The access models developed will generally do well to predict vulnerability of pregnant and postpartum mothers seeking health care in facilities, though the extent to which such vulnerabilities are emphasized differ by geographical location. The study rides on a rich spatially granular surveillance dataset and goes a step further in accounting for geographical access impedances than mainstream access models using travel distance alone.

### Limitations

It is important to understand similarities and trends between the two access determination techniques in order to strengthen public health policy formulation. This will best be achieved with the least methodological and data limitations in the analysis. This study however, had several methodological limitations and therefore findings should be used within the confines of this limitation. The current models under consideration did not account for other environmental (natural, built, or social) variables, which should be included for a more comprehensive and therefore realistic model. However, the focus on precipitation in this study is motivated by its relative contribution to impeding access, compared with other environmental factors [5] and its inclusion in this study is a step forward to what is found in mainstream modelling of access to care. The importance of precipitation in impeding access may not be necessarily consistent across different spatial groupings and is therefore also subject to geographic scrutiny. The scope of the current study is limited in the spatial variability in consistence of importance of precipitation in impeding access.

Samples in this study may be spatially non-random and therefore, spatial inferences drawn from the findings should take this into consideration. The Bland–Altman method of analysis in methods comparison performed best when the range of absolute values is narrow, and the absolute differences are small. However, for the samples under study there were a few instances where the absolute differences were very large violating the validity of the assumptions for this comparative technique.

The choice to use a median paired difference in preference to a mean paired difference is informed by the occurrence of instances of outliers in the data under analysis, in which case the median score has been proven less sensitive to outliers.

### Implications

Travel time to care facilities is not associated with uptake of services. These results do not imply that increasing geographic accessibility to care reduces health service uptake, but rather points to the fact that access is not only a function of travel time to facilities, but also a function of other factors such as quality of care offered in facilities. Therefore, interventions targeting a reduction in maternal and neonatal mortality should consider a holistic approach in the package of interventions.

These findings are important to health policy makers who seek to obtain local information on access to maternal health care. However, assumptions in modelling access across different geographies should be treated with care taking into consideration geographical and socio-economic variations between geographies. The analysis provides evidence that there is need for careful considerations of other factors that impede access.

## Data Availability

All the street data that was used in this project can be available to anyone upon request. The precipitation data is available from FEWSNET (ftp://cpc.ncep.noaa.gov/fews/fewsdata/africa/arc2/geotiff/). The CLIP data is not available in the public domain as per the provisions of UBC PREEMPT data handling protocols but may be available under special clearances for use of the data.

## Footnotes

1 P = Potential access (modelled geographical access), R = realised access/self-reported travel time, A = travel time to the facility where care was sought by the woman.

2 CLIP facility surveillance collected data at facilities for women reporting to facilities whereas regular surveillance was the routine data collection on each woman during the CLIP trials. All this data is centrally managed from the University of British Columbia, Canada.

3 Solving a route analysis means finding the quickest route between two points in a network, and this analysis depends on the impedance chosen to solve for. In this analysis the impedance is time, and the best route is the quickest route. Hence, the best route can be defined as the route that has the lowest impedance, or least cost.
